# One-stage surgical management for lumber brucella spondylitis with anterior debridement, autogenous graft, and instrumentation

**DOI:** 10.1097/MD.0000000000011704

**Published:** 2018-07-27

**Authors:** Xin Hua Yin, Zhong Kai Liu, Bao Rong He, Ding Jun Hao

**Affiliations:** Department of Spine Surgery, Hong Hui Hospital, Xi’an Jiaotong University College of Medicine, Xi’an, P.R. China.

**Keywords:** anterior, debridement, lumber brucella spondylitis, surgery

## Abstract

Literature reporting on lumber brucella spondylitis (LBS) is rare, therefore, the purpose of this study was to evaluate the outcomes of one-stage surgical management for lumber brucella spondylitis by anterior debridement, autogenous grafts, and instrumentation. This was a retrospective cohort study including 16 patients with lumber brucella spondylitis by treated from January 2009 to October 2011 in our department. All cases underwent one-stage anterior internal fixation, debridement, and bone fusion; clinical and radiographic results were analyzed and compared. All patients were followed up for an average of 35.3 ± 8.1 months (range, 24–48 months). Brucella spondylitis was completely cured in all patients with bone fusion achieved in 4.8 ± 1.3 months. Visual analog scale (VAS) scores were significantly improved between the preoperative and last follow-up visit and neurological function classification showed significant improvement after surgical intervention. Preoperatively, the Cobb angle was 20.7 ± 9.8°, and measured 8.1 ± 1.3° at the last follow-up visit. The outcomes of follow-up demonstrated that one-stage surgical treatment with anterior debridement, fusion, and instrumentation can be an effective and feasible treatment method for lumber brucella spondylitis.

## 1 Introduction

Brucellosis is a common zoonotic infection caused by facultative intracellular bacteria of the genus *Brucella*. Infection with this pathogen affects the entire body, and a frequent osteoarticular complications of *Brucella* infection is spondylitis.^[1]^ The lumbar segment is by far the most commonly affected structure in patients with spondylitis, followed by the dorsal and cervical segments.^[2]^ Brucella spondylitis (BS) usually develops in the upper endplate due to localization of strong blood flow; the lower endplate can also be involved.^[3]^ Methods for the treatment of lumber brucella spondylitis (LBS) are still controversial, and vary from only chemotherapy to a combination of antimicrobial treatment and surgery. Antimicrobial chemotherapy is the mainstay of brucella spondylitis treatment, but it has been found to be ineffective in preventing cases of progressive kyphosis deformity, unbearable continuous backache, late onset paralysis, and refractory disease.^[4–6]^ To our knowledge, anterior surgical treatment of LBS has rarely been reported, therefore, we report our results regarding the surgical treatment of LBS by one-stage anterior debridement, autogenous grafts, and instrumentation.

## 2 Methods

This study was approved by the Ethics Committee of the Honghui Hospital, Xi’an Jiaotong University Health Science Center, and written informed consent was obtained from all patients. Between January 2009 to October 2011, 16 patients with a diagnosis of lumber brucella spondylitis underwent anterior surgery at our hospital. The cohort was comprised of 12 men and 4 women, averaging 45.0 ± 10.3 years old (range, 29–67), receiving a minimum 2-year follow-up postoperatively. All cases presented with constitutional symptoms such as back pain, weakness, malaise, and intermittent fever (≥38.5 °C, between afternoon and evening) with weight loss. American Spinal Injury Association (ASIA) classification was used to assess compromised neurological function. In total, 11 patients demonstrated neurological deficits (ASIA B to D). Clinical outcomes were assessed preoperatively and at the last follow-up visit using visual analog scale (VAS) questionnaire. Additionally, the lumber Cobb angle was measured by making 2 imaginary lines—one along the top surface of the immediate upper normal vertebral body, and the other away from the diseased segment. The average lumber Cobb angle was 20.7 ± 9.8° (range, 18–35°). Finally, a diagnosis of lumber brucella spondylitis was based on positive blood culture, positive bacterial culture of a biopsy specimen, or a titer >1:160 on the brucellosis standard tube agglutination test (STAT); furthermore diagnosis was confirmed with clinical follow-up.^[7]^ The exclusion criteria of this study were: previous surgery; lumbosacral lesion induced by disease such as tuberculosis, metastasis or multiple myeloma; and patients with poor health status (Table 1).

**Table 1:**
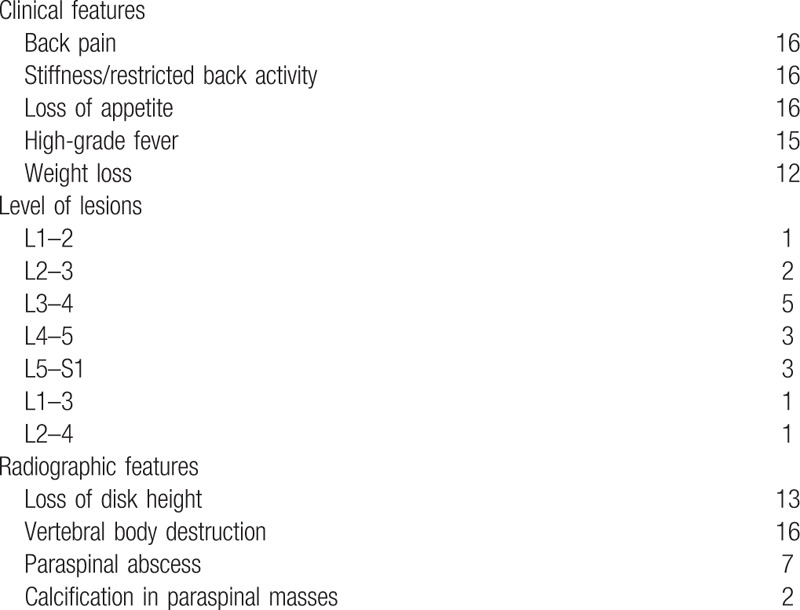
Clinical data of patients.

### 2.1 Preoperative preparation

Once a diagnosis of brucellosis was confirmed, patients were given anti-brucellosis drugs, doxycycline (200 mg/d, orally), rifampicin (450 mg/d, orally), and streptomycin (1 g/d, intramuscularly) prior to surgery. Nutrition enhancement and correction of anemia and hypoproteinemia were also carried out. When the patient had a normal or significantly decreased body temperature and anemia as well as hypoproteinemia had resolved, surgery was carried out.

### 2.2 Surgical procedure

**Figure 1:**
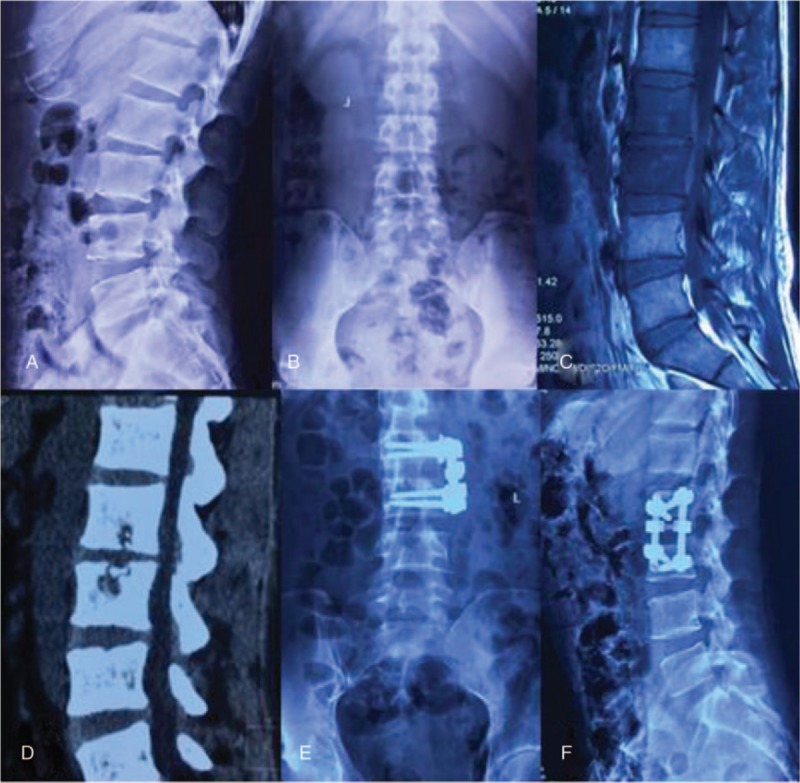
Case 1: A 29-year-old man with brucella spondylitis at L2–3. (A–D): The lateral and anteroposterior x-ray, MRI, and CT showed the destruction of vertebral bodies of L2–3. (E and F) The lateral and anteroposterior view of x-ray showed that the anterior infected site had healed and bony union was achieved at the final follow-up. CT = computed tomography; MRI = magnetic resonance imaging.

The patients were operated on in the lateral position after administration of general endotracheal anesthesia. Usually, a standard anterolateral approach to the spine via a retroperitoneal flank incision was employed, the same as the anterolateral approach of spine tuberculosis. After exposure, any pus was drained and granulomatous tissue and/or necrotic material were debrided. Radical debridement was performed using a rongeur and curette until sclerosing bone was completely removed and healthy, bleeding margins were obtained. After sufficient spinal cord decompression, correction of the kyphosis was performed by distracting between adjacent, normal vertebrae. Then, strut grafts were implanted to reconstruct the anterior column and restore normal height. Finally, anterior instrumentation was performed and the debrided material was sent for histopathologic examination as well as antibiotic sensitivity testing (Fig. 1).

### 2.3 Postoperative management

After surgery, a drainage tube was removed when drainage flow became clear and less than 30 mL/24 h. In this study, 16 patients received first line therapy for brucellosis which is recommended by the World Health Organization: tetracycline, 500 mg every 6 hours orally or doxycycline, 100 mg every 12 hours orally for 6 weeks with an aminoglycoside (streptomycin, 1 g/d intramuscularly for 2–3 weeks or gentamicin 5 mg/kg/d intravenously or intramuscularly for 7–10 days). Early ambulation was permitted 7.4 ± 1.5 days after the surgery in all patients. Orthoses were worn at least 3 months postoperatively and all patients were examined clinically and radiologically at 3, 6, and 12 months after surgery and then once a year. X-ray, blood test, erythrocyte sedimentation rate (ESR) were examined. Bone grafting fusion was assessed using the modified radiologic criteria of Lee et al.^[8]^ Additionally, preoperative, postoperative, and final follow-up changes in degree of kyphotic deformity and neurologic status were noted. All statistical analyses were conducted using SPSS 20.0 software (SPSS, Inc., Chicago, IL) and a paired Student *t* test was performed to compare parameters both pre- and postoperatively as well as at the final follow-up. *P*-value <.05 was considered to be statistically significant.

## 3 Results

The average follow-up was 35.3 ± 8.1 months (range, 24–48 months). The mean duration of surgery was 237.4 ± 29.5 minutes and the mean amount of bleeding was 580.2 ± 140.3 mL. Postoperatively, all cases had significant improvement in constitutional symptoms and back pain. However, there were 2 cases of postoperative complications: 1 case of wound infection and 1 case of pain at the graft harvesting site. No perioperative complications related to instrumentation or decompression were reported. The grafted bones were fused in 4.8 ± 1.3 months in all patients. Antibody titer against STAT, ESR were normal in 16 patients. On last follow-up, neurological recovery was observed in 11 patients with previous neurological deficit; specifically, 5 cases with grade D deficits and 4 cases with grade C recovered to grade E and 2 cases with grade B improved to grade D. VAS scores were significantly improved at last follow-up and the average lumber Cobb angle was significantly decreased at 7.5 ± 1.5° (range, 6–9.2°) postoperatively and had resolved to 8.1 ± 1.3° (range, 7–10°) at last follow-up. This result was statistically significant different compared with the preoperative lumber Cobb angle (*P* < .05), but had there was no significant difference between the postoperative angle and that at final follow-up (*P* < .05) (Tables 2–3).

**Table 2:**
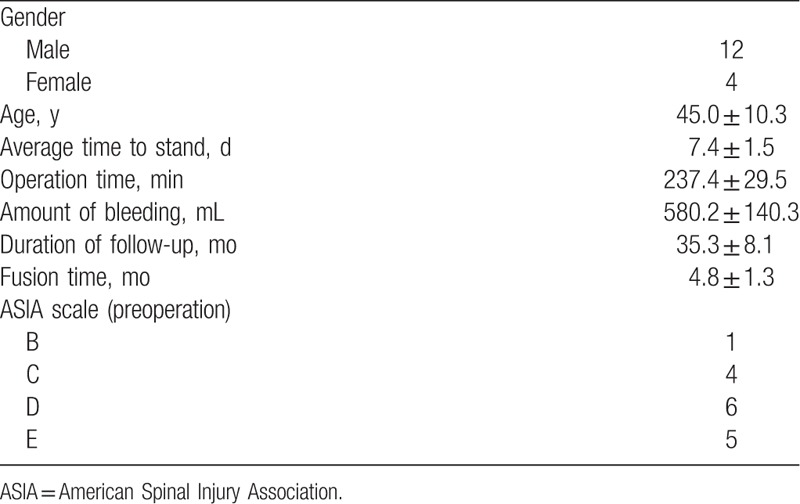
Clinical data on the surgery.

**Table 3:**
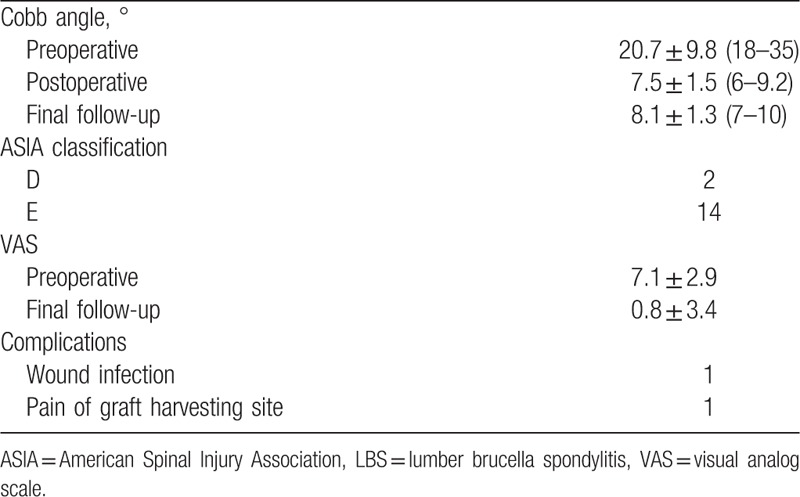
Clinical outcomes of surgical treatments for LBS.

## 4 Discussion

Brucellosis is a bacterial zoonosis with a high degree of morbidity contributing to the economic problems of many developing countries.^[9–11]^ The spine, especially the lumber is the predilection site of brucellosis, accounts for 6% to 12% of all series and is the foremost cause of the debilitating and disabling complications.^[12–15]^ Combinations of antimicrobial chemotherapy remains the mainstay of treatment of BS. While most of BS patients can be cured by conservative treatment,^[16–18]^ it is common to find residual kyphosis and instability at the end of treatment.

In a multicenter, retrospective and comparative study involving a total of 293 patients with BS, most of cases were cure by antimicrobial chemotherapy and surgery was performed in 32 patients. This study demonstrated that patients who fail conservative treatment, or experience a compressing effect from an inflammatory mass or abscess, or suffer from spinal instability, progressive spinal collapse, or neurological deficits, surgical treatment is frequently imperative.^[19]^

Various methods of surgical treatment in patients with brucella spondylitis have been reported. In 2001, Nas et al^[20]^ reported on 11 BS patients who were first treated with a combination of antibiotics. Further surgery was required for 8 patients. Two patients underwent microsurgical decompression and discectomy and 6 patients had decompression laminectomies and discectomies. All patients were considered cured at last follow-up. It should be noted that debridement of BS lesions or drainage of such abscesses, causes spine instability, inducing a loss of lordosis and the formation of kyphosis deformity. Therefore, surgery does not having a long-term advantage over ambulant chemotherapy alone. In addition, because debridement was limited, the long-term therapeutic effect of surgery is affected and internal fixation may be required.

There is a minority literature referring to the surgical treatment of LBS for the reconstruction of spinal stability and prevention of complications. Some authors have expressed their concerns about the risk of using instrumentation in setting spinal infection because this may decrease antibiotic effectiveness while increasing bacterial adherence and glycocalyx formation. Notably, Oga et al^[21]^ evaluated the adherence properties of *Staphylococcus epidermidis* to stainless steel and found that the bacteria heavily colonized the rods. However, Chang and Merritt^[22]^ considered titanium to be less prone to bacterial colonization compared with other materials such as polymethyl-methacrylate and stainless steel. Recently several publications have confirmed the safety and efficacy of this instrumentation in treatment of spinal infections, provided there is complete debridement of infected tissue and an appropriate course of antibiotic therapy is adhered to by the patient.^[22–25]^

Infected tissues should be thoroughly debrided and the abscesses drained extending back to healthy bleeding bone to allow for subsequent tissue healing. The objective of debridement is to accelerate the natural course of healing by evacuating the bulk of the infected tissue and opening vascular channels from the subchondral bone. This outcome facilitates antibiotic delivery and accelerates the invasion process by the reparative granulation tissues. After extensive debridement of the infected tissue, structural bone, or cage grafting may be performed simultaneously. In fact, an anterior approach provides the best access for debridement and stabilization for grafting and facilitates rehabilitation. For these reasons anterior radical surgery has become the standard operative treatment when surgical intervention is essential.

In 2016, Chen et al^[26]^ reported on 24 consecutive patients with BS who underwent a one-stage operation, between 2012 and 2014, combining debridement, autogenous bone graft, and instrumentation with a posterior approach. In the 24 patients, there was no recurrence of BS and VAS scores and neurologic function were significantly improved. Although good results obtained in this study, it has been suggested that removing a tuberculosis (TB) focus using the posterior approach in spinal surgery could cause intraspinal infection and central nervous system complications from TB infection, such as TB meningitis.^[27,28]^ In addition, this approach fails to create enough operating space for complete removal of the focus, and it is thus likely to injure the spinal cord or nerve root in the operation. This approach tends to cause spinal stability due to resection of the side of the facet joint.

Kemp et al^[29]^ found that anterior spinal fusion permits more rapid bony healing with less vertebral column collapse, faster patient rehabilitation, and a less incidence infection reactivation compared with nonoperative treatment. Additionally, Muckley et al^[30]^ reported on 3 patients treated with radical decompression and debridement with interbody fusion and anterior spinal fixation with no recurrence of infection or loss of reduction during follow-up. In 2007, Lee et al^[31]^ retrospectively analyzed of 32 patients (23 with pyogenic spondylitis, 8 with spine tuberculosis, and 1 with fungal spondylitis) who underwent surgical treatment. In total, 26 out of 32 cases underwent one-stage anterior resection, interbody autografting, and instrumentation. The average follow-up period was 33.4 months and most of patients were cured.

Many studies have also shown good results in cases of spine tuberculosis or infection who underwent anterior surgery. Thus, researchers have begun to explore the feasibility of one-stage surgical management for lumber brucella spondylitis by anterior debridement, decompression and autogenous rib grafts, and instrumentation. In our cohort, we have observed rapid improvements in all 16 cases with one-stage surgery. Significant improvements of the lumber Cobb angle were observed from 20.7 ± 9.8° preoperatively to 8.1 ± 1.3° at last follow up. All 16 patients exhibited satisfactory bone fusion at 4.8 ± 1.3 months thus demonstrating that anterior debridement, decompression and autogenous rib grafts, and instrumentation are a safe and effective procedure for the treatment of LBS.

## 5 Conclusion

In our series, we find that the indications of anterior surgery on LBS are as follows: local back pain unrelieved by conservative treatment, abscess formation, suffering from spinal instability or progressive collapse, a compressing effect of the inflammatory mass or abscess with considerable neurologic deficit, no posterior column involvement. To our knowledge this study is unique because of the rarity of reports on anterior surgical treatment for LBS. Our research used a retrospective cohort study to evaluate the outcomes of surgical management of lumber brucella spondylitis via an anterior approach. By recording the correction rate, neurologic status, operation time, blood loss, VAS, and fusion time, we found that one-stage anterior surgery might be a safe and effective approach for the treatment of lumber brucella spondylitis.

## Author contributions

**Conceptualization:** Xinhua Yin.

**Data curation:** Baorong He.

**Writing – original draft:** Dingjun Hao.

**Writing – review and editing:** Zhongkai Liu.
